# P-2133. Clinic evaluation and validation of a visual loop-mediated isothermal amplification assay for a fast detection of *Aspergillus spp*

**DOI:** 10.1093/ofid/ofae631.2288

**Published:** 2025-01-29

**Authors:** José Nicolás Aguirre Pineda, Hansel Hugo Chávez Morales, Carlos Flores Nunez, Mario Alberto Mujica Sánchez, Durán Barrón Martha Angella, Ivan Hiram H Aguilar Zuñiga

**Affiliations:** Instituto Nacional de Enfermedades Respiratorias-México (INER), Cuidad de México, Distrito Federal, Mexico; Instituto Nacional de Enfermedades Respiratorias-México (INER), Cuidad de México, Distrito Federal, Mexico; INER, MEXICO CITY, Distrito Federal, Mexico; Instituto Nacional de Enfermedades Respiratorias-México (INER), Cuidad de México, Distrito Federal, Mexico; Instituto Nacional de Enfermedades Respiratorias-México (INER), Cuidad de México, Distrito Federal, Mexico; Instituto Nacional de Enfermedades Respiratorias-México (INER), Cuidad de México, Distrito Federal, Mexico

## Abstract

**Background:**

Aspergillus is one of the most frequent causes of fungal infections with a high mortality. Due to the low specificity of the clinical manifestations, the lack of pathognomonic radiological findings, and the low sensitivity and relative slowness of microbiological methods, the diagnosis of Aspergillus remains a challenge. The molecular diagnostic tools enable rapid detection of a wide variety of infectious agents but requires expensive equipment and highly qualified personnel. Such disadvantages could be avoided by LAMP. This study aimed to determine the diagnostic performance of a visual LAMP assay for rapid detection of Aspergillus spp.

**Figure:**
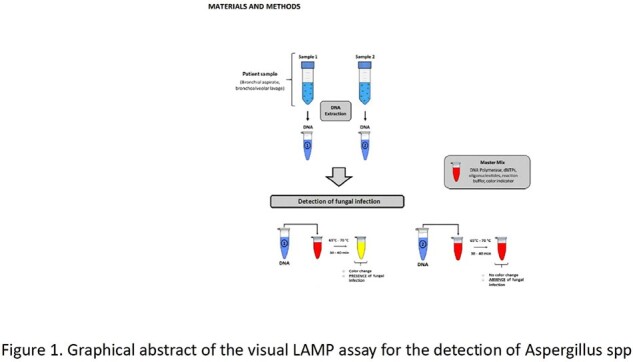

**Methods:**

The PCR-LAMP design was carried out, the molecular detection of the fungus was done through the amplification of a fragment of the low-evolution genes 18S and 28S. The amplification of the target genes was performed using the Bst 2.0 DNA polymerase enzyme, included in the WarmStart® Colorimetric LAMP 2X Master Mix (DNA & RNA) kit from NEB. Once the total DNA had been extracted and purified, the reaction mixture was prepared and incubated at 65°C for 30-40 minutes. Detection of amplification of the target regions was identified by eye, positive reactions had a color change from pink to yellow (Figure 1)

The assay was validated by processing bronchoalveolar lavages and bronchial aspirates from patients with a positive galactomannan antigen test and isolation of Aspergillus spp. by culture and classified according to the EOCRT criteria.

**Table 1.**
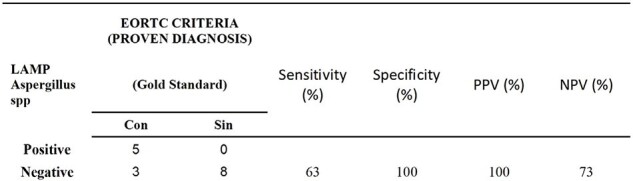
Comparison between the performance of PCR LAMP compared to PROVEN DIAGNOSIS. NPV: Negative predictive value; PPV: positive predictive value.

**Results:**

Of the 65 samples analyzed in total. 15 samples were reported positive by PCR LAMP and the rest were negative for Aspergillus spp. For the detection of Aspergillus spp, the sensitivity by means of the molecular test was 63% and the specificity found was 82%. 57 patient samples included for analysis met the EOCRT criteria. According to the EORTC diagnostic criteria, cases identified as possible and probable IPE predominated. The sensitivity of Aspergillus LAMP in patients with proven IFI criteria was 63%, with a specificity of 100%. A lower sensitivity was obtained in patients with proven and possible IF, 20% and 21% respectively.

**Table 2.**
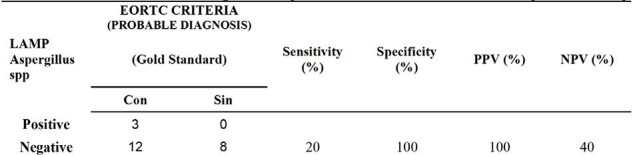
Comparison between the performance of PCR LAMP compared PROBABLE DIAGNOSIS. NPV: Negative predictive value; PPV: positive predictive value.

**Conclusion:**

Despite the low performance, a method was obtained that can be used at the point of care, fast and easy to use, to detect fungal infections.

**Table 3.**
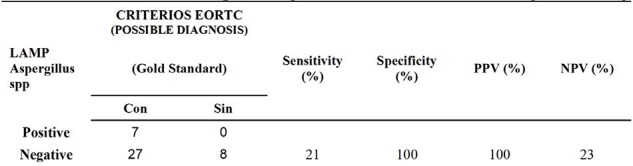
Comparison between the performance of PCR LAMP compared POSSIBLE DIAGNOSIS. NPV: Negative predictive value; PPV: positive predictive value.

**Disclosures:**

José Nicolás Aguirre Pineda, n/a, Pfizer: Grant/Research Support Hansel Hugo Chávez Morales, n/a, Pfizer: Grant/Research Support Carlos Flores Nunez, PHD, Pfizer: Grant/Research Support Mario Alberto Mujica Sánchez, n/a, Pfizer: Grant/Research Support

